# Influence of Detonation Spraying Parameters on the Microstructure and Mechanical Properties of Hydroxyapatite Coatings

**DOI:** 10.3390/ma17215390

**Published:** 2024-11-04

**Authors:** Zhuldyz Sagdoldina, Marcin Kot, Daryn Baizhan, Dastan Buitkenov, Laila Sulyubayeva

**Affiliations:** 1 Research Center «Surface Engineering and Tribology», Sarsen Amanzholov East Kazakhstan University, Ust-Kamenogorsk 070000, Kazakhstan; zh.sagdoldina@gmail.com (Z.S.); buitkenovd@gmail.com (D.B.); lsulyubayeva@gmail.com (L.S.); 2 Research School of Physical and Chemical Sciences, Shakarim University, Semey 071412, Kazakhstan; 3 Faculty of Mechanical Engineering and Robotics, AGH University of Krakow, 30-059 Krakow, Poland; kotmarc@agh.edu.pl

**Keywords:** titanium, biomedical coatings, detonation spraying, hydroxyapatite, surface properties

## Abstract

The process of osteointegration depends significantly on the surface roughness, structure, chemical composition, and mechanical characteristics of the coating. In this regard, an important direction in the development of medical materials is the development of new techniques of surface modification and the creation of bioactive ceramic coatings. Calcium-phosphate materials based on hydroxyapatite have been proposed as bioactive ceramic coatings on titanium implants for the effective acceleration of bone tissue healing. To obtain bioactive ceramic coatings, pulse power sources are best suited, namely detonation spraying, in which the energy of the explosion of gas mixtures is used as a source of pulse action. The pulse mode of operation in the detonation spraying method is preferable for the formation of bioactive ceramic coatings. It provides a high velocity of hydroxyapatite particles, which promotes their effective fixation on the titanium substrate, while minimizing the heating of the material. This approach preserves the substrate structure and improves the coating adhesion. Four different types of coatings with varying O_2_/C_2_H_2_ molar ratios, ranging from 2.6 to 3.7, were obtained using detonation spraying. Powders and obtained coatings of hydroxyapatite were studied by Raman spectroscopy and XRD structural analysis. The results of XRD phase analysis showed the partial conversion of the hydroxyapatite phase to the α-tricalcium phosphate (α-TCP) phase during the detonation spraying process. The results obtained by Raman spectroscopy indicate that hydroxyapatite is the main phase in coatings. All hydroxyapatite-based coatings exhibited hydrophobic properties, which was confirmed by contact-angle values above 90° in wettability tests, characteristic of hydrophobic surfaces. The adhesive strength of the coatings was measured by the scratch test method. Tribological tests were conducted using the ball-on-disk method under both dry conditions and in Ringer’s solution. This approach enabled the evaluation of wear resistance and friction coefficient of the coatings in different environments, simulating both lubrication-free conditions and those resembling physiological environments.

## 1. Introduction

Implants used in traumatology, orthopedics, and maxillofacial surgery are characterized by complexity and labor-intensive production [1]. This is due to the fact that the implant material must fulfil many: bending and fatigue strength, flexibility, and the ability to create complex shapes, which significantly narrows the range of available materials. Currently, titanium and its alloys are widely used as bearing materials for dental and orthopedic implants due to their suitable mechanical properties and high corrosion resistance [2,3,4,5,6,7,8]. However, titanium alloys are bioinert materials because there is no direct chemical bond between them and bone tissue after implantation [9]. A significant difference in the elasticity modulus with bone tissue can lead to bone destruction near the implant as a result of fretting wear induced by shear stress and inflammatory processes as well as osteoporosis during long-term implantation.

A number of technological approaches have now been proposed for implant surface modification [10,11,12,13,14,15]. These include surface activation and coatings to improve the adhesion of new bone tissue to the implant and enhance implant osseointegration [16,17]. In this regard, the application of calcium phosphate coatings on titanium implants has been proposed as a promising method to accelerate bone healing [18,19,20]. These coatings not only ensure the functioning of implants in the living body environment, but also promote integration with its tissues [21]. One of the most important materials widely used for this purpose is hydroxyapatite (HA).

The main biological advantage of HA coatings is to enhance bone formation, accelerate binding between the implant surface and surrounding tissue and reducing the potentially harmful release of metal ions. It also creates strong bonds at the interface with titanium implants, possibly due to the presence of some chemical bonding between hydroxyapatite and the titanium substrate.

In recent decades, bioactive coating techniques for implants have developed significantly due to their key role in improving the osseointegration and biocompatibility of implants. Nowadays, there are many methods of hydroxyapatite coating application, each with its own peculiarities and advantages depending on the final application. Among the most commonly used methods are micro-arc oxidation [22], magnetron sputtering [23], the formation of composite polymer coatings [24], and vacuum-arc sputtering with ion assisted spraying [25]. Micro-arc oxidation is an electrochemical process that allows the creation of porous oxide layers with high adhesive properties on the surface of metal implants [26]. This method is especially effective for creating bioactive surfaces with a fine grain structure, which contributes to improved osteointegration. Magnetron sputtering, in turn, allows for thin, dense coatings with high homogeneity to be obtained, which is critical for controlling the coating thickness and structure [27].

In addition to the above methods, others of interest include plasma spraying, high velocity oxygen fuel spraying (HVOF), and detonation spraying. These methods are characterized by their high rate of material deposition on the substrate and the ability to control the coating microstructure [28]. Among them, detonation spraying may be preferable due to its ability to create coatings with high density and adhesion strength to the implant surface as well as the cost of deposition. The detonation spraying process is based on the explosion of a combustible gas mixture that accelerates hydroxyapatite particles to hypersonic speeds. This leads to the fact that the particles not only penetrate into the substrate, but also form a coating with high adhesion and very low porosity [29]. Such coatings have high mechanical properties, which makes them promising for medical applications, especially in the field of implantology. A comparison of different methods of bioactive coatings, such as magnetron sputtering, micro-arc oxidation, and detonation spraying, shows that each of them has unique advantages. However, due to its characteristics, detonation spraying occupies an important place among technologies aimed at improving the osseointegration and durability of medical implants.

## 2. Materials and Methods

**Figure 1:**
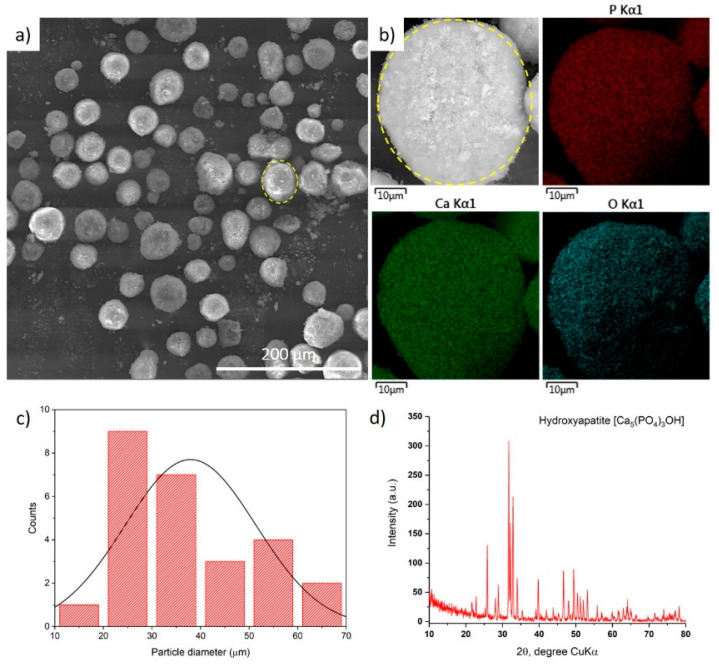
(**a**) SEM image with (**b**) EDS spectrum of hydroxyapatite powder; (**c**) particle size distribution of feedstock powder; (**d**) XRD analysis of hydroxyapatite powder.

The coatings were applied to Grade 2 titanium and pre-treated by sandblasting with electro corundum powder with a particle size of 100–200 μm at an air pressure of 0.4–0.5 MPa. After that, the substrate was rinsed with distilled water and air-dried. Commercially available hydroxyapatite (HA) powder provided by Medicoat, Mägenwil, Switzerland was selected to obtain coatings. The hydroxyapatite powder has an average particle size of about 37.5 μm and is characterized by a predominantly spherical particle morphology, as presented in the SEM images (Figure 1a,b). The spherical shape of the particles favors their uniform distribution and tight packing on the substrate surface during the spraying process, which is important for the formation of a durable and homogeneous coating. Energy dispersive spectral analysis (EDS) was carried out to determine the elements present in the hydroxyapatite powder and their relative ratio. As can be seen in the image (Figure 1b), the hydroxyapatite powder contained three major elements: calcium (Ca), phosphorus (P), and oxygen (O). These elements are characteristic for the chemical structure of Ca_5_(PO_4_)_3_OH hydroxyapatite. As expected, the Ca/P ratio corresponded to the theoretical value of 1.67, confirming the high quality of the powder and its suitability for use in medical coatings. The granulometric composition of the raw material powder was determined from the SEM image using ImageJ software version 1.54g (Figure 1c). In addition, XRD analysis showed that the powder was synthetic hydroxyapatite (Figure 1d), indicating its purity and stability.

**Figure 2:**
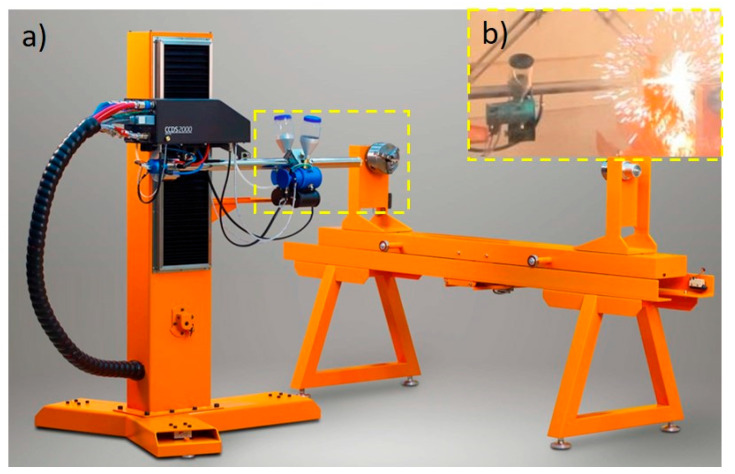
Detonation spraying device: (**a**) detonation complex CCDS2000; (**b**) coating application process.

During the study, four different types of coating were produced using the detonation spraying method. Three samples were produced for each coating type, which ensured the repeatability and reliability of the results. The coating process was carried out using a CCDS2000 detonation device (LIH SB RAS, Novosibirsk, Russia) (Figure 2) controlled by a computer system that allows for precise control of the spraying parameters such as the oxygen to acetylene ratio and explosive charge volume [30]. This ensures high accuracy and reproducibility of the coating [31]. The use of an automatic control system ensured stable detonation spraying conditions, which is critical for obtaining homogeneous and durable hydroxyapatite coatings with high adhesion properties.

In the detonation spraying process, a mixture of combustible gases is fed into a chamber and detonated by a spark plug. The detonation creates a shock wave that accelerates the particles to supersonic speeds, so that the particles penetrate deeply into the surface of the material to form a strong and dense coating with high adhesion properties [32].

In this study, a detonation gun with a barrel length of 450 mm and diameter of 26 mm was used to apply the hydroxyapatite (HA) coatings. Acetylene (C_2_H_2_) and oxygen (O_2_) were used as the combustible component and oxidizing agent, respectively. Before each shot, the system was purged with nitrogen, which was also used to feed the powders through the powder feeder. The chemical composition of the detonation products and spraying efficiency depend on the oxygen to acetylene ratio (O_2_/C_2_H_2_), which varied from 2.6 to 3.7 depending on the type of coating, as listed in Table 1. The key parameter affecting the detonation spraying process is the fraction of the barrel volume filled with the acetylene-oxygen gas mixture. This parameter, called ‘explosive charge’, ranged from 73% to 78% of the barrel volume in this study. This parameter has a direct influence on the detonation energy and, consequently, on the quality and structure of the coating obtained by spraying.

The structure of the obtained samples was investigated by Raman spectroscopy on an AFM-Raman Solver Spectrum, manufactured by NT-MDT (Zelenograd, Moscow, Russia). A blue laser with a wavelength of 473 nm and a maximum power of 35 mW was used to excite the vibrational modes. The laser radiation was focused using a lens with a magnification of 100×, which provided a spot size on the sample surface of the order of 2 × 10^−6^ m. Spectra were obtained during the measurements and then processed using the Savitzky–Golay method to smooth the data and remove noise. A 2nd-order polynomial was used for processing, which ensured high accuracy and minimized data distortion. The Raman shift had an absolute uncertainty equal to 4 cm^−1^, indicating high accuracy and reliability of the data obtained, which is necessary to correctly analyze the fine structural features of the samples and determine the phase composition or possible impurities. The samples were also subjected to X-ray phase analysis using an XRD-6000 diffractometer (Shimadzu Corporation, Kyoto, Japan) with monochromatic emission of a copper anode (CuKα) with a wavelength of 1.54056 Å. The following parameters were set for the measurements: acceleration voltage 45 kV, beam current 30 mA, scanning step 0.02° in the range of 2θ angles from 20° to 80°, and the signal accumulation time at each step of 0.5 s. The PDF4^+^ database and POWDER CELL 2.4 full profile analysis software were used to analyze the phase composition. Surface wettability was determined by measuring the contact angle using the sitting drop method. An OCA 15EC video angle meter was used for this purpose. All measurements were carried out at a constant ambient temperature of 25 °C. Distilled water was used as the polar liquid. For each sample, 10 drops of liquid were applied using an auto-feeding micropipette. After each drop was applied, a photograph of the surface was taken and exported to a computer for further analysis. The contact angles for all 10 drops were calculated and averaged to give the average contact angle for each sample. The surface roughness (*S_a_*) was examined using a non-contact 3D profilometer, MICROMEASURE 3D station (STIL SAS, Aix-en-Provence, France). The surface morphology was studied using a scanning electron microscope (SEM) (MIRA3 LMU, TESCAN, Brno, Czech Republic) at a magnification of ×1000.

The mechanical properties of the hydroxyapatite coatings were investigated using the nanoindentation method with an NHT3 nanoindenter (Anton Parr company, Graz, Austria). Hardness tests were performed for a maximum loading force of 20 mN. The Oliver and Pharr procedure was used for the hardness and elasticity modulus calculations [33]. The tests were conducted using a three-sided Berkovich pyramid. In accordance with the method proposed by Oliver and Pharr [33], the hardness H was calculated using the following relationship:

(1) $$H=\frac{P_{max}}{A}$$

where *P_max_* is the maximum applied load, and *A* is the contact area between the indenter and the sample.

The elastic modulus of the samples was determined from the slope of the unloading curve using the following equations [33]:

(2) $$E_{r}=\frac{\sqrt{\pi }\cdot S}{2\cdot \sqrt{A}}$$

where *E_r_* is the reduced modulus of elasticity:

(3) $$\frac{1}{E_{r}}=\frac{1-v^{2}}{E}+\frac{1-v_{i}^{2}}{E_{i}}$$

*E*, *ν* are the Young’s modulus and Poisson’s ratio of the tested material; *E_i_*, *ν_i_* are the Young’s modulus and Poisson’s ratio of the indenter material; *S* is the contact stiffness; *A* is the contact area taking into account permanent deformation.

The measurement of the adhesion strength of coatings was carried out using the scratch test method. Scratch tests were performed for a linearly increasing force from 0 up to 30 N, with the test length of 5 mm.

Tribological tests were conducted using a T-21 tribotester in accordance with the ISO 20808:2016 standard [34]. The experiments were carried out under sliding friction conditions in a “ball-on-disk” configuration with rotational motion. The tests were conducted over 50,000 cycles with a normal force of 2 N, a friction radius of 5 mm, and a rotational speed of 0.1 m/s, using an Al_2_O_3_ ball with a diameter of 6 mm as the counter body. The following formula was used to calculate the volumetric wear:

(4) $$W_{v}=\frac{V}{F_{n}\cdot s}\left[\frac{mm^{3}}{N\cdot m}\right]$$

where is *V* the volume of worn material [mm^3^]; *F_n_* is the normal force exerting pressure on the sample [N]; *s* is the friction path [m].

The surface profile measurements of the samples after the wear tests were performed with a ProFilm 3D non-contact interferometric profilometer.

The second tribological test of the samples was conducted on a TRB3 tribometer using the standard “ball-on-disk” method (ASTM G 133-95 [35]) in Ringer’s solution, which simulates the body’s fluid. The chemical composition of Ringer’s solution is 9.0 g/L NaCl, 0.42 g/L KCl, 0.48 g/L CaCl_2_, and 0.2 g/L NaHCO_3_. A ceramic ball made of silicon nitride (Si_3_N_4_) with a diameter of 6 mm was selected as the counter body. The test conditions included a normal load of 6 N, a sliding speed of 3 cm/s, and total sliding of 5000 cycles.

## 3. Results and Discussion

**Figure 3:**
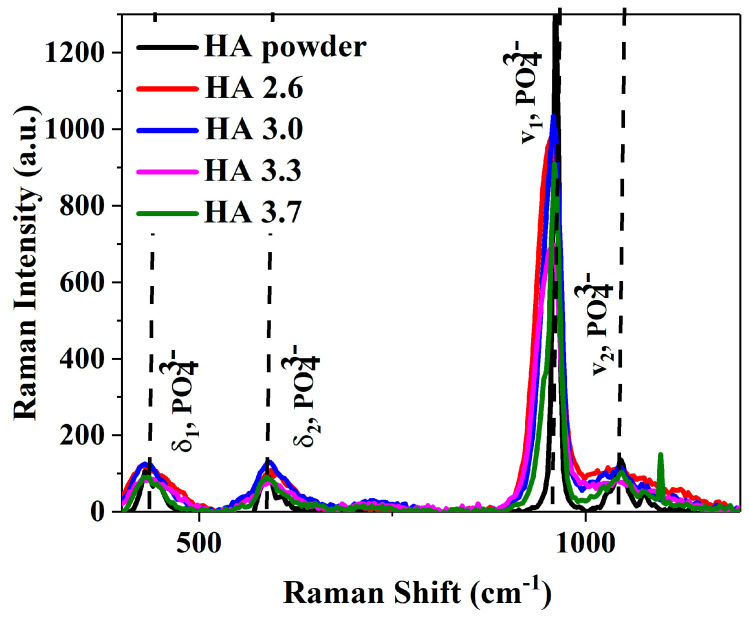
Raman spectra of Ti-implant coated HA at different O_2_/C_2_H_2_ ratios.

**Figure 4:**
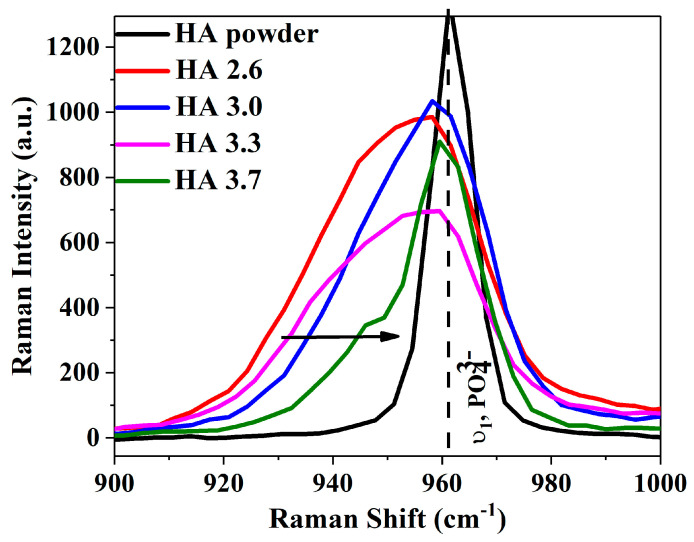
The close-up region centered around v_1_, the symmetric phosphate (PO_4_^3−^) vibration bond.

The results present the effects of varying O_2_/C_2_H_2_ ratios (2.6, 3.0, 3.3, and 3.7) on the hydroxyapatite (HA) coatings deposited by the detonation technique on titanium (Ti) substrates on the microstructure of coatings as well as on their mechanical properties. The Raman spectra of HA-coated Ti implants at these different O_2_/C_2_H_2_ ratios are shown in Figure 3, along with a reference sample of purely crystalline HA. A detailed view of the region centered at 962 cm^−1^ (Figure 4) corresponded to the symmetric phosphate stretch (v_1_). The Raman spectra displayed distinct vibrational modes of HA including δ_1_ (symmetric bending between 430 and 450 cm^−1^), δ_2_ (asymmetric bending around 600 cm^−1^), v_1_ (symmetric stretch near 963 cm^−1^), and v_2_ (asymmetric stretching between 1000 and 1100 cm^−1^). Table 2 lists the observed Raman peaks across the different O_2_/C_2_H_2_ ratios along with their vibrational assignments [36]. The phosphate (PO_4_^3−^) vibrational modes, particularly the v_1_ mode, are essential in determining the structural state of the material. The v_1_ mode is highly sensitive to crystallinity, typically manifesting as a sharp peak in highly crystalline HA. Any shifts or broadening on this peak signal indicate the presence of structural defects or phase transitions.

For the crystalline HA sample, the v_1_ mode appeared at 962 cm^−1^, with a narrow full width at half maximum (FWHM) of 9.24 cm^−1^, indicating a pure and well-ordered structure (black solid curve—Figure 4). However, when HA was coated onto Ti implants at varying O_2_/C_2_H_2_ ratios, significant changes in the v_1_ mode occurred, reflecting the formation of structural defects. At an O_2_/C_2_H_2_ ratio of 2.6, the v_1_ peak shifted from 962 cm^−1^ to 958 cm^−1^, with a substantial broadening of the FWHM to 33.54 cm^−1^, suggesting considerable structural disorder. At a ratio of 3.0, the v_1_ peak remained at 958 cm^−1^, but the FWHM narrowed to 27.72 cm^−1^, indicating a slight improvement in crystallinity, probably due to the increased oxygen content during the detonation process, which facilitates better conditions for HA formation [13]. As the O_2_/C_2_H_2_ ratio increased to 3.3, the FWHM broadened further to 35.58 cm^−1^, indicating the introduction of additional structural defects and the onset of an amorphous phase. A previous study [13] suggested that increasing the O_2_/C_2_H_2_ ratio within a certain range could intensify the impact of detonated gases on the sprayed particles. This increased dynamic effect affects the particles’ motion and behavior upon impact, which, in turn, influences the resulting microstructure of the coating. Such changes are observed as variations in the full width at half maximum (FWHM) value in X-ray diffraction (XRD) measurements, indicating alterations in the material’s crystalline structure or residual stress state. At the highest ratio of 3.7, the v_1_ peak shifted slightly to 960 cm^−1^ with an FWHM of 19.04 cm^−1^, suggesting an improved crystalline structure. The excess oxygen during the detonation process probably causes thermal fluctuations and rapid cooling, leading to FWHM changes. The amorphous structure is characterized by a higher density of surface defects and loss of long-range crystalline order [37]. This may be beneficial for applications requiring rapid bone resorption and regeneration [38]. Table 3 provides a comprehensive summary of the Raman shifts and FWHM values observed for each sample. Here, the sample with the O_2_/C_2_H_2_ ratio of 3.7 seems to be the best choice for titanium (Ti) dental implants exposed to high mechanical loads. The high crystallinity suggests a greater structural stability, lower defect density, and better overall durability, making it sufficient for permanent implants that require long-term functionality and osteointegration.

**Figure 5:**
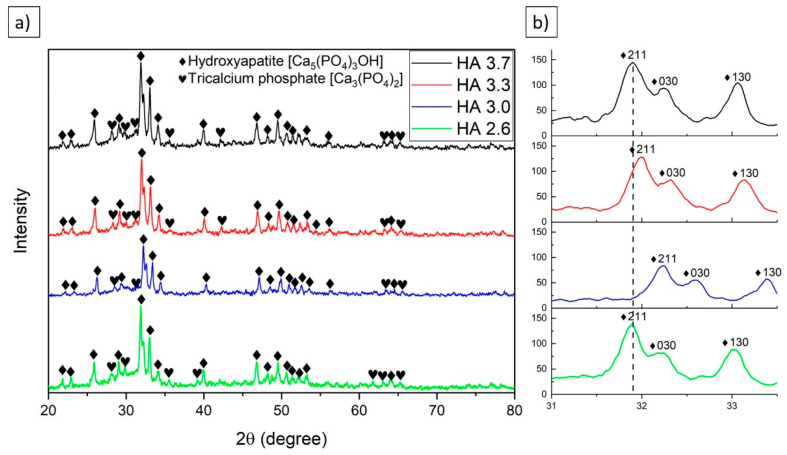
X-ray diffraction patterns (**a**) and enlarged sections (**b**) for hydroxyapatite coatings obtained at different O_2_/C_2_H_2_ ratios.

Figure 5 shows the results of the X-ray phase analysis of the obtained hydroxyapatite (HA) coatings. The main phase of the coating was Ca_5_(PO_4_)_3_OH hydroxyapatite, but, at higher temperatures, its partial decomposition was observed to form α-TCP tricalcium phosphate (α-Ca_3_(PO_4_)_2_). As is known from the literature [39,40], the decomposition of hydroxyapatite occurs when the critical temperature, about 1650 °C, is reached, accompanied by dehydroxylation and the loss of crystal structure. Hydroxyapatite has a relatively high melting point, but during detonation spraying, intensive thermal processes occur, which can lead to partial destruction of the hydroxyapatite structure. When critical temperatures are reached, HA decomposes into tricalcium phosphate (α-TCP at 1200 °C) and other calcium-phosphate phases, such as tetracalcium phosphate (TTCP), which may be present in negligible amounts [41]. It is important to note that the process temperature and particle cooling rate play a key role in the phase composition of the final coating. The phase composition of hydroxyapatite coatings has a significant influence on their biological and mechanical properties. Hydroxyapatite is known for its biocompatible properties and promotes implant osseointegration. However, phases such as tricalcium phosphate (TCP) can affect coating solubility and biodegradation. In particular, α-TCP and β-TCP are bioresorbable phases that can dissolve in biological fluids over time, promoting the gradual release of calcium and phosphate, which can stimulate bone regeneration [42].

Figure 5 shows the X-ray diffraction patterns, in which a slight shift in the 2θ peak positions could be observed for different O_2_/C_2_H_2_ ratios. This shift may be related to changes in the crystal structure of hydroxyapatite (HA), indicating several possible factors. The (211) peaks shifted to the left as the O_2_/C_2_H_2_ ratio increased, which may suggest minor changes in the lattice parameters. This is caused by changes in the interplanar spacings, which could result from the inclusion or substitution of elements in the crystal structure. As the O_2_/C_2_H_2_ ratio increases, the peak shifts become more noticeable, possibly indicating improved crystallinity or a reduction in material defects. If the peaks shift toward lower 2θ values, this usually indicates an increase in lattice parameters, which could be due to the inclusion of elements with larger atomic radii or the presence of internal stresses. However, the main phase remains hydroxyapatite, despite these minor structural changes. The peak shifts suggest potential stress in the crystal structure or changes in chemical composition, which influence the mechanical and physical properties of the coatings.

Table 4 presents the detailed XRD analysis results for the coatings synthesized at different O_2_/C_2_H_2_ ratios. It includes data on the detected phases, phase content (expressed in mass percentages), lattice parameters, crystallite size, and relative change in lattice spacing (Δd/d × 10^−3^). The analysis primarily focused on the two key calcium phosphate phases: hydroxyapatite (Ca_5_(PO_4_)_3_OH) and tricalcium phosphate (Ca_3_(PO_4_)_2_). Hydroxyapatite, which has a specific chemical composition of Ca_5_(PO_4_)_3_OH, possesses a hexagonal crystal structure with a space group of P63/m. The lattice parameters for hydroxyapatite across the samples ranged from a = 0.939 nm to a = 0.941 nm for the “a” axis and c = 0.688 nm to c = 0.690 nm for the “c” axis. The lattice parameters for hydroxyapatite across the 290 samples ranged from a = 0.93896 nm to a = 0.94128 nm for the ‘a’ axis and c = 0.68811 nm to c = 0.69045 nm for the ‘c’ axis. These parameters are close to the theoretical values of a = b = 0.9432 nm and c = 0.6881 nm for pure hydroxyapatite, indicating good crystallinity in the coating. Crystallite size for the hydroxyapatite phase varied between 48 nm and 62 nm, with the sample at an O_2_/C_2_H_2_ ratio of 3.7 showing the largest crystallites, suggesting potential growth and better crystalline order with higher O_2_ content. The second phase, tricalcium phosphate (Ca_3_(PO_4_)_2_), also showed variation in the lattice parameters across the samples, with values for the ‘a’ axis ranging from 1.28684 nm to 1.29870 nm, and for the ‘b’ axis from 2.72012 nm to 2.73800 nm. The crystallite size of this phase varied from 18 nm to 25 nm. The relative change in lattice spacing (Δd/d × 10^−3^) indicates some degree of lattice distortion, with the highest distortion observed in the Ca_3_(PO_4_)_2_ phase at the O_2_/C_2_H_2_ ratio of 3.7, which could be attributed to the incorporation of secondary elements or structural defects. These findings indicate that the composition and structural properties of the coatings vary depending on the O_2_/C_2_H_2_ ratio, with higher oxygen content promoting larger crystallite sizes and a more ordered crystal structure in both hydroxyapatite and tricalcium phosphate phases. This suggests a strong influence of the gas ratio on the phase composition and crystallinity, which is crucial for optimizing the coating properties in biomedical applications such as improved adhesion and durability.

**Figure 6:**
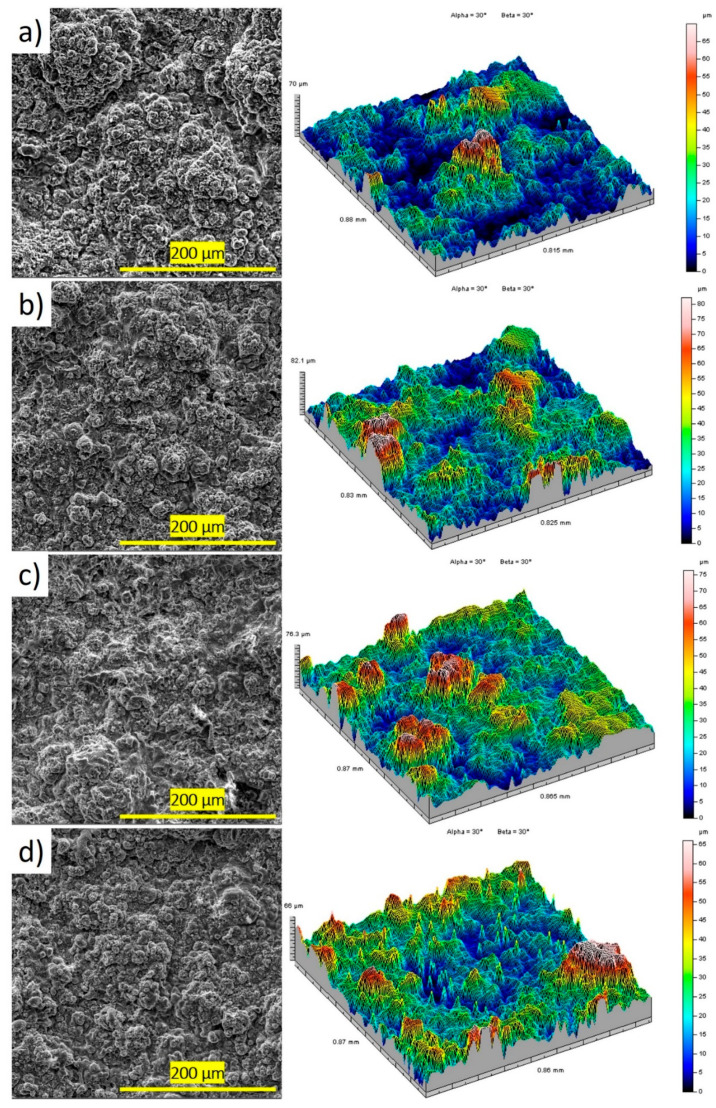
SEM images and 3D surface roughness profiles of (**a**) HA 2.6, (**b**) HA 3.0, (**c**) HA 3.3, and (**d**) HA 3.7 coatings.

Figure 6 shows the SEM images of the surface and 3-D microphotographs of the surfaces of the tested hydroxyapatite coating. As a result of the analysis, it was found that the coating surface had a heterogeneous structure with the presence of pores and was characterized by a layered and undulating arrangement of structural components [43]. A small number of microspherical and dune-like particles as well as well-flattened splats were observed on the coating surface. Due to insufficient heating of large powder particles (≥45 μm), their core did not fully melt, which led to the formation of several areas with irregularities and uneven shapes on the surface. This was attributed to the temperature and short flight time of the powder particles during detonation spraying. More specifically, the shell of these particles melted, but the core remained intact [44], resulting in a heterogeneous microstructure with highly developed surface topography, which favors better interaction with bone, necessary in biomedical applications such as implant coatings. The roughness parameters for the coating samples showed the following values: the arithmetic mean surface height (S_a_) for the HA 2.6 sample was 7.9 μm, while it increased to 9.5–11 μm for other coatings. The values of the S_q_, S_p_, and S_v_ parameters also varied between samples, confirming the heterogeneous structure of the coating. The roughness parameters are summarized in Table 5.

**Figure 7:**
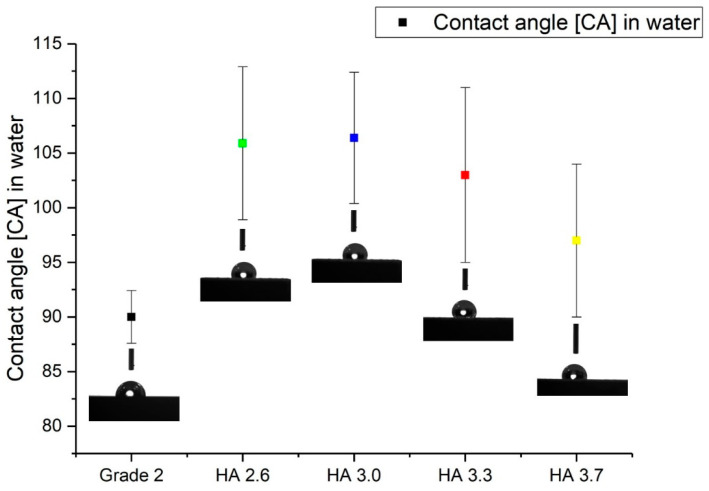
Contact angles of water on the surface of the coatings.

To evaluate the hydrophobic properties of the obtained coatings, contact-angle measurements were carried out using distilled water. The results of these measurements are presented in Figure 7. As can be seen, all hydroxyapatite-based coatings exhibited hydrophobic behavior, as evidenced by contact-angle values above 90° [45]. The highest contact angle of 106.4° was found for the HA 3.0 coating, indicating the pronounced hydrophobic properties of this coating [46]. This may be due to its specific microstructure and surface texture resulting from the detonation spraying process. A high contact angle indicates low water wettability, which may be useful in some biomedical applications where it is necessary to prevent excessive cell or protein adhesion on the implant surface. The smallest contact-angle was measured for the pure Grade 2 titanium substrate, being 90.5°. This result confirms that the titanium substrate itself is less hydrophobic compared to the hydroxyapatite-based coatings [31]. The other coatings showed intermediate contact-angle values ranging between 96.8° and 106.4°, also confirming their hydrophobic properties. As the molar ratio of oxygen/acetylene (O_2_/C_2_H_2_) increases, the hydrophobicity of the coatings also varies due to changes in the coating microstructure and roughness. These factors may play an important role in biomedical applications, especially in the context of osseointegration and interaction of the implant surface with biological fluids [47].

**Figure 8:**
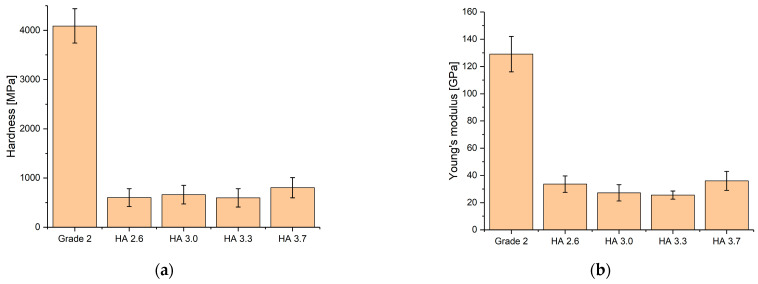
Nanohardness (**a**) and modulus of elasticity (**b**) of coatings and titanium.

The nanohardness and elasticity modulus values of the coatings are presented in Figure 8. One of the key requirements for the surface of bone implants is that the elastic modulus and hardness of the artificial material should closely match those of bone tissue: *H* = 2–4 GPa and *E* = 7–26 GPa [40]. The maximum nanohardness value was achieved for the HA 3.7 coating (803 ± 4.8 MPa). The reason for this is the higher amount of crystalline hydroxyapatite (HA) in the HA3.7 coatings compared to the other coatings with lower hardness values (600 ± 60 MPa). Accordingly, it can be assumed that the mechanical properties of the HA 3.7 coating, exhibiting noticeably higher hardness and elastic modulus values, surpass those of the other coatings. This highlights the important role of hydroxyapatite phases in the coatings compared to the crystalline phases of tricalcium phosphate (TCP). Trzaskowska et al. [48] highlight that the crystalline structure of hydroxyapatite (HA) plays a crucial role in enhancing its mechanical properties. They emphasize that the sintering temperature has a significant impact on the material’s microstructure, which in turn affects its mechanical characteristics such as strength and biocompatibility. The proper choice of sintering temperature allows for the optimization of HA’s properties, making it more suitable for use in medical implants and other biomaterials.

**Figure 9:**
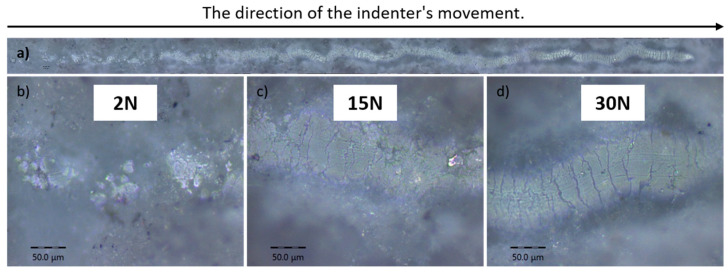
The optical images of the adhesion tracks of the coatings obtained at the O_2_/C_2_H_2_ ratio of 2.6. Subfigure (**a**) shows an overview of the entire track created by the indenter’s movement, indicating the direction of motion (left to right). Images (**b**–**d**) indicate the load applied to the indenter.

The optical images of the scratches on the coatings obtained at the O_2_/C_2_H_2_ ratio of 2.6 are presented in Figure 9, illustrating the progression of coating damage with the increase in applied load. Figure 9a shows a general view of the scratch along the entire trajectory of the indenter’s movement. Enlarged sections are shown in Figure 9b, Figure 9c, and Figure 9d, where the load on the indenter was 2 N, 15 N, and 30 N, respectively. The HA 2.6 coating demonstrated better cohesion, with the first cracks appearing at 15 N, compared to the other coatings.

**Figure 10:**
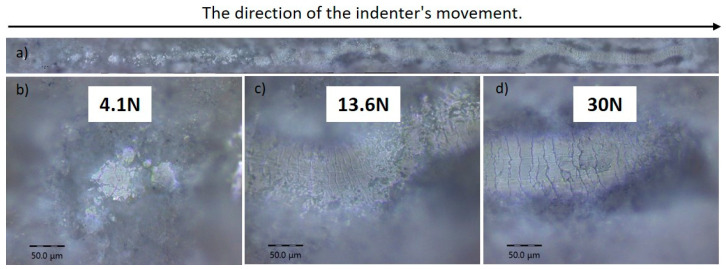
The optical images of the adhesion tracks of the coatings obtained at the O_2_/C_2_H_2_ ratio of 3.0. Subfigure (**a**) shows an overview of the entire track created by the indenter’s movement, indicating the direction of motion (left to right). Images (**b**–**d**) indicate the load applied to the indenter.

Figure 10 presents the results of the scratch test for a coating deposited at an O_2_/C_2_H_2_ ratio of 3.0. In Figure 10a, the general trajectory of the indenter is shown as it moves across the surface of the coating under increasing load. Enlarged images of sections with varying loads are shown in Figure 10b, Figure 10c, and Figure 10d, corresponding to forces of 4.1 N, 13.6 N, and 30 N, respectively. At a load of 4.1 N (Figure 10b), no visible damage to the coating was observed, indicating its resistance to small forces. The first signs of material degradation, such as cracks, appeared at a load of 13.6 N (Figure 10c), marking the onset of critical damage. As the load increased further to 30 N (Figure 10d), significant surface destruction with multiple cracks became evident, indicating progressive damage to the coating. Despite this high load, no adhesive cracks or coating delamination were observed. These findings are important for determining the critical load at which the coating begins to lose its protective properties.

**Figure 11:**
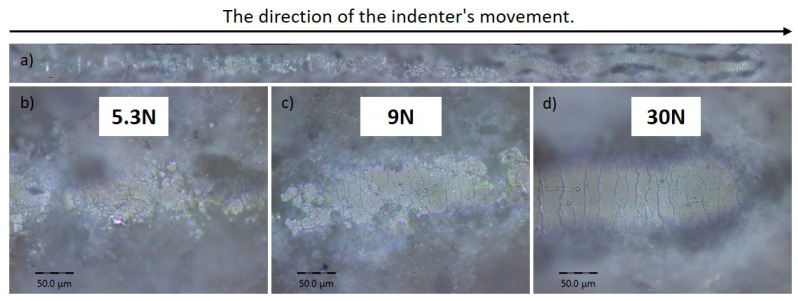
The optical images of the adhesion tracks of the coatings obtained at the O_2_/C_2_H_2_ ratio of 3.3. Subfigure (**a**) shows an overview of the entire track created by the indenter’s movement, indicating the direction of motion (left to right). Images (**b**–**d**) indicate the load applied to the indenter.

The image shows the results of a scratch test for a coating formed by detonation spraying at an O_2_/C_2_H_2_ ratio of 3.3. Figure 11a demonstrates the overall track left by the indenter as it moved across the surface under a progressively increasing load. Enlarged sections under loads of 5.3 N, 9 N, and 30 N are shown in Figure 11b, Figure 11c, and Figure 11d, respectively. At 5.3 N (Figure 11b), minor coating damage was observed, but cracks became apparent at 9 N (Figure 11c), indicating the onset of material degradation.

**Figure 12:**
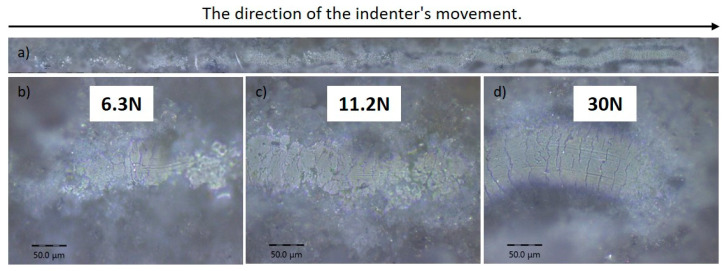
The optical images of the adhesion tracks of the coatings obtained at the O_2_/C_2_H_2_ ratio of 3.7. Subfigure (**a**) shows an overview of the entire track created by the indenter’s movement, indicating the direction of motion (left to right). Images (**b**–**d**) indicate the load applied to the indenter.

Figure 12 presents the results of the scratch test for a coating produced by the detonation spraying method at an O_2_/C_2_H_2_ ratio of 3.7. In Figure 12a, the overall track left by the indenter is shown as it moved across the coating surface under increasing load. Figure 12b, Figure 12c, and Figure 12d shows enlarged sections of the track at loads of 6.3 N, 11.2 N, and 30 N, respectively. At a load of 6.3 N, initial damage to the coating was observed, while at 11.2 N, cracks became more noticeable, indicating the onset of material structure failure. At a load of 30 N, Figure 12d shows significant damage to the coating, with the development of multiple cracks, indicating progressive degradation of adhesion. These results suggest that the critical load for the onset of coating failure lies between 6.3 N and 11.2 N.

At an O_2_/C_2_H_2_ ratio of 3.7, there was an increase in the proportion of crystalline hydroxyapatite and the size of the crystallites, which negatively affected the material’s cohesive properties. Under such conditions, large crystals become less plastic compared to smaller crystallites or amorphous regions, leading to stress concentration zones. This is especially evident in mechanical tests, such as scratch tests, where large crystallites distribute the load unevenly. As a result, microcracks form in the material, weakening its cohesive properties. The larger and harder the crystallites at this O_2_/C_2_H_2_ ratio, the higher the likelihood of material failure under external loads, as the structure becomes less capable of evenly distributing stress. This leads to a reduction in the mechanical stability of the coating and its faster degradation under increasing loads. Thus, at an O_2_/C_2_H_2_ ratio of 3.7, the large number of large crystallites negatively impact the strength and durability of the coating.

The absence of titanium substrate exposure during the scratch test in all coatings can be explained by the high strength and cohesive properties of the coatings themselves. This means that the coating is not only well-bonded to the titanium surface, but also has internal resistance, allowing it to withstand significant mechanical loads without peeling or breaking down to the substrate level. This is probably due to a high-quality application process that ensured not only sufficient thickness but also uniformity of the coating, minimizing defects and porosity. Additionally, such behavior may indicate good adhesion of the coating to the substrate, providing high resistance to mechanical impact. The optimal phase ratio and coating structure enable the efficient distribution and absorption of mechanical stresses, preventing stress concentration in individual areas, ultimately reducing the risk of destruction and the exposure of the titanium substrate. This may also indicate that the coating material has high fracture toughness, preventing the formation of large defects during mechanical testing. These observations enable the assessment of the coating’s strength limits and its resistance to mechanical loads, which is an important indicator for its potential application in real-world conditions.

**Figure 13:**
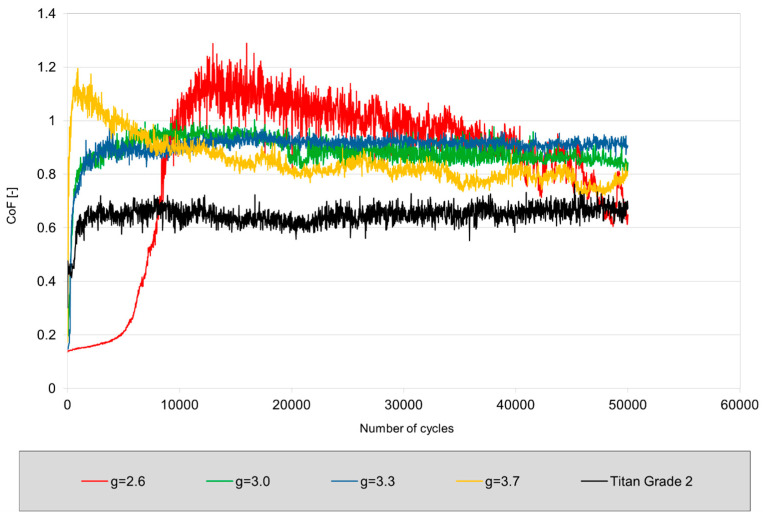
Dependence of the coefficient of friction (CoF) on the number of cycles for coatings obtained at different O_2_/C_2_H_2_ ratios and pure titanium (Grade 2).

Based on the graph and table, the tribological behavior of the coatings produced at different O_2_/C_2_H_2_ ratios could be further analyzed and compared with pure titanium (Grade 2). The Figure 13 clearly shows that the coatings with O_2_/C_2_H_2_ ratios of 3.0 and 3.3 exhibited the most stable coefficient of friction throughout the test (up to 50,000 cycles), indicating good wear resistance. The coating with a ratio of 2.6 showed a sharp increase in the coefficient of friction after 8000 cycles, indicating rapid wear and loss of functionality at early stages of use. The low coefficient of friction at the initial stage may be related to the heterogeneous microstructure with a highly developed surface relief. Such a structure helps to reduce the contact area between surfaces, which leads to a decrease in friction force. As the number of friction cycles increases, the coating’s microstructure may gradually adapt, smooth out, or degrade on a microlevel. As a result of this process, the coefficient of friction further decreases as the surface becomes more stable and smoother. The coating with a ratio of 3.7 also showed relatively stable behavior, but with a higher coefficient of friction, which may suggest less effective anti-friction properties. Although tribological properties may not be as important for dental implants, for other applications such as joint prostheses or orthopedic implants, tribological characteristics are critically important. In these cases, minimizing friction and wear becomes essential for extending the lifespan of implants and ensuring their stable operation under constant mechanical loads. It is also important to reduce the generation of wear particles as they can lead to inflammation or implant rejection.

**Figure 14:**
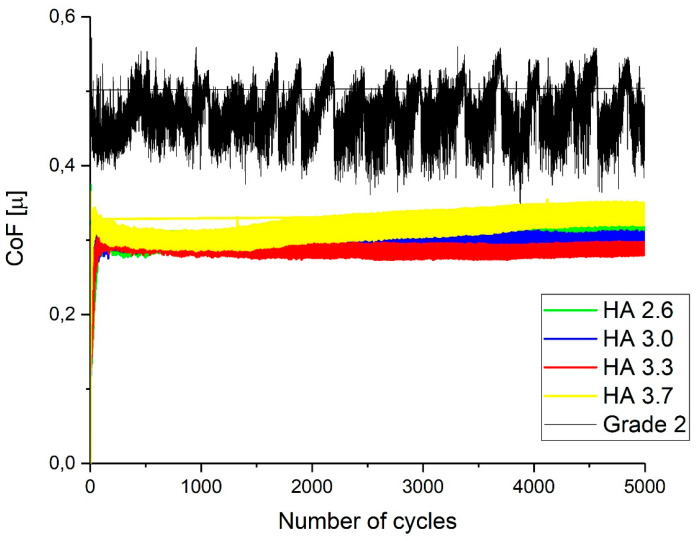
Dependence of the coefficient of friction (CoF) on the number of cycles for coatings in Ringer’s solution obtained at different ratios of O_2_/C_2_H_2_ and pure titanium (Grade 2).

Figure 14 presents the results of the tribological tests of the hydroxyapatite coatings and titanium (Grade 2) in Ringer’s solution. The graph shows the dependence of the coefficient of friction (CoF) on the number of cycles for each coating and titanium, allowing for an assessment of their wear resistance in conditions simulating a physiological environment. Grade 2 titanium exhibited the highest and most unstable coefficient of friction, indicating faster wear and a deterioration in the mechanical properties under friction. In contrast, the hydroxyapatite coatings, particularly at the O_2_/C_2_H_2_ ratios of 3.0 and 3.3, demonstrated much more stable and lower CoF values throughout the test, indicating better wear resistance. Additionally, the micrographs showed wear marks on the surfaces of the coatings and titanium, where the surfaces of the hydroxyapatite coatings retained their integrity better than titanium, further confirming their advantage in such conditions. These data underscore the importance of using hydroxyapatite coatings to enhance the durability and wear resistance of biomedical implants, especially when exposed to physiological fluids such as Ringer’s solution.

**Figure 15:**
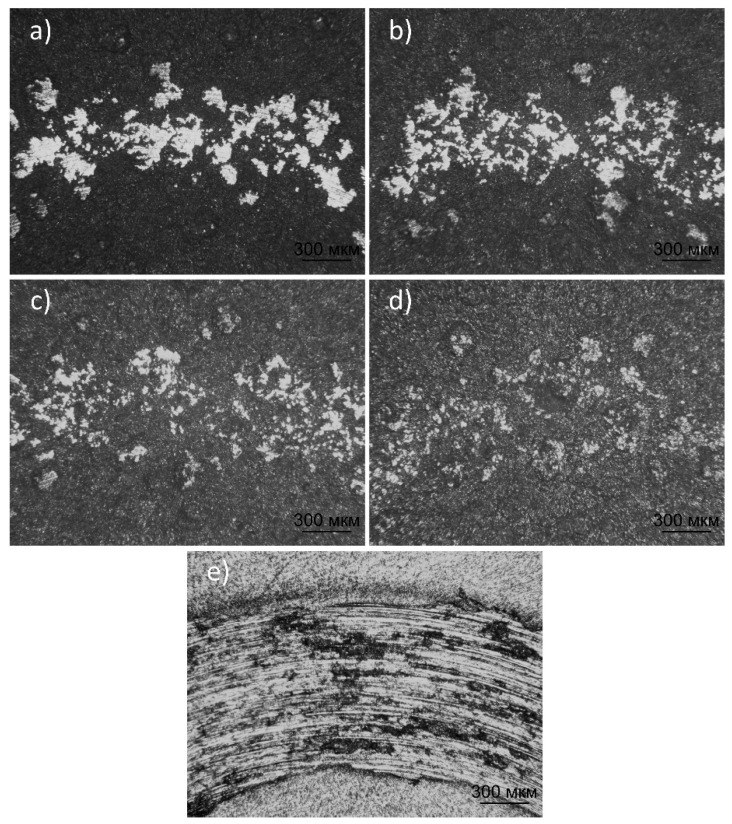
Micrographs of traces of wear of the hydroxyapatite coating obtained at various O_2_/C_2_H_2_ ratios: (**a**) HA 2.6; (**b**) HA 3.0; (**c**) HA 3.3; (**d**) HA 3.7, and (**e**) Grade 2.

Figure 15 shows the typical wear morphology of the hydroxyapatite and titanium (Grade 2) coatings after tribological testing in Ringer’s solution. The presented micrographs illustrate the differences in the degree of wear of hydroxyapatite and titanium (Grade 2) coatings following the tribological tests. The coating produced at an O_2_/C_2_H_2_ ratio of 2.6 exhibited significant damage with large areas of spalling, as confirmed by the increased friction coefficient on the graph, indicating low wear resistance. The coating with an O_2_/C_2_H_2_ ratio of 3.0 showed less damage and smoother wear, reflected in the lower friction coefficient, suggesting better mechanical durability. The coating with an O_2_/C_2_H_2_ ratio of 3.3 showed an even lower and more stable friction coefficient, corresponding to more uniform wear and minimal damage visible on the micrographs. The O_2_/C_2_H_2_ 3.7 coating also demonstrated minimal wear marks and good surface integrity, as confirmed by the low friction coefficient on the graph. Meanwhile, titanium (Grade 2) exhibited the highest level of wear, with deep wear tracks on the surface, which corresponded to the highest and most unstable friction coefficient, indicating insufficient wear resistance. These results highlight that hydroxyapatite coatings, especially at the O_2_/C_2_H_2_ ratios of 3.0 and 3.3, significantly outperform titanium in terms of wear resistance, making them more suitable for biomedical implant applications where durability in friction conditions is crucial.

The data in Table 6 demonstrate the tribological characteristics of various materials including coatings with different O_2_/C_2_H_2_ ratios and pure titanium (Grade 2) in both dry conditions and Ringer’s solution. The coating with an O_2_/C_2_H_2_ ratio of 2.6 in dry conditions showed the lowest coefficient of friction (CoF) of 0.5, but had the highest wear volume among the coatings of 741 × 10^−6^ mm^3^/Nm, indicating insufficient durability. The coating with an O_2_/C_2_H_2_ ratio of 3.0 exhibited a moderate coefficient of friction of 0.87 and a relatively low wear volume of 422 × 10^−6^ mm^3^/Nm, indicating a good balance between friction and wear resistance. The coating with an O_2_/C_2_H_2_ ratio of 3.3 had the lowest wear volume among all materials of 331 × 10^−6^ mm^3^/Nm, with a coefficient of friction of 0.90, indicating its high wear resistance. The coating with an O_2_/C_2_H_2_ ratio of 3.7 had a relatively low coefficient of friction of 0.85 but the highest wear volume of 1230 × 10^−6^ mm^3^/Nm, indicating poor wear resistance. Pure titanium (Grade 2) showed a coefficient of friction of 0.65 and a wear volume of 734 × 10^−6^ mm^3^/Nm, making it inferior to the coatings with ratios of 3.0 and 3.3 in terms of wear resistance. In Ringer’s solution, the coating with an O_2_/C_2_H_2_ ratio of 2.6 demonstrated a low coefficient of friction of 0.302 and a moderate wear volume of 150 × 10^−6^ mm^3^/Nm, indicating decent characteristics in this environment. The coating with an O_2_/C_2_H_2_ ratio of 3.0 had the lowest coefficient of friction of 0.287 and one of the lowest wear volumes of 113 × 10^−6^ mm^3^/Nm, making it one of the most effective coatings in Ringer’s solution. The coating with an O_2_/C_2_H_2_ ratio of 3.3 showed a low coefficient of friction of 0.296 and the smallest wear volume of 108 × 10^−6^ mm^3^/Nm, confirming its excellent wear-resistant properties in the solution. The coating with an O_2_/C_2_H_2_ ratio of 3.7 demonstrated a slightly higher coefficient of friction of 0.319 and a relatively high wear volume of 152 × 10^−6^ mm^3^/Nm, indicating less effective tribological characteristics compared to other coatings. Pure titanium had the highest coefficient of friction of 0.471 and a wear volume of 630 × 10^−6^ mm^3^/Nm, making it the least suitable material for use in such conditions. The data indicate that coatings with the O_2_/C_2_H_2_ ratios of 3.0 and 3.3 provided the best tribological properties in both dry conditions and Ringer’s solution. They exhibited an excellent balance of low friction and minimal wear, making them the most advantageous for applications where wear resistance and minimal friction are important.

## 4. Conclusions

The study of hydroxyapatite coatings obtained by detonation spraying shows that the O_2_/C_2_H_2_ ratio significantly affects the crystallinity, structural integrity, and mechanical properties of hydroxyapatite (HA) coatings on titanium implants. The sample with an O_2_/C_2_H_2_ ratio of 3.7 demonstrated the highest crystallinity with a narrow FWHM value of 19.04 cm^−1^, low defect density, and better structural stability. This was confirmed by the XRD results, as an increase in oxygen content led to crystallite growth and improved crystalline order, especially at an O_2_/C_2_H_2_ ratio of 3.7. Coatings obtained at the O_2_/C_2_H_2_ ratios of 2.6, 3.0, and 3.3 also demonstrated good mechanical properties, but with lower hardness and elasticity modulus values compared to the sample with an O_2_/C_2_H_2_ ratio of 3.7 (803 ± 4.8 MPa), which is associated with a higher content of crystalline hydroxyapatite. However, the large crystallites that formed at this ratio led to a deterioration in cohesive properties, as indicated by the appearance of microcracks and reduced strength under increasing loads. The coating with an O_2_/C_2_H_2_ ratio of 2.6 showed a hardness of about 600 MPa but exhibited better cohesive properties, as cracks were formed during scratch testing at a 15 N load, indicating good fracture resistance. At the O_2_/C_2_H_2_ ratios of 3.0 and 3.3, the hardness and elastic modulus were higher than in the 2.6 sample, but a decrease in cohesion was observed, and cracks appeared at lower loads (9 and 13.6 N, respectively). Tribological studies showed that hydroxyapatite coatings with the O_2_/C_2_H_2_ ratios of 3.0 and 3.3 exhibited the best tribological properties both in dry conditions and Ringer’s solution. These coatings demonstrate a stable and low coefficient of friction as well as low wear index and seem to be optimal for applications where wear resistance and low friction are crucial. In contrast, the coating with an O_2_/C_2_H_2_ ratio of 2.6 showed rapid wear and loss of functionality after a certain number of cycles, while the coating with an O_2_/C_2_H_2_ ratio of 3.7, despite having a relatively low coefficient of friction, demonstrated poorer wear resistance. Hydroxyapatite coatings significantly outperform pure titanium (Grade 2) in terms of wear resistance and friction stability, especially when exposed to physiological fluids. Thus, hydroxyapatite coatings with the O_2_/C_2_H_2_ ratios of 3.0 and 3.3 are the most promising for use in biomedical implants, especially at high contact stresses and in contact with biological fluids.

## Figures and Tables

**Table 1:** Detonation spraying parameters of the HA powder.

Sample	Molar Ratio O_2_/C_2_H_2_	Explosive Charge, % (Volume of Barrel Filled with Gas)
HA 2.6	2.6	73
HA 3.0	3.0	74
HA 3.3	3.3	77
HA 3.7	3.7	78

**Table 2:** The assigned Raman shift at different O_2_/C_2_H_2_ ratios.

Raman Shift Frequency (cm^−1^)	Assignments
423–429	(PO_4_)^3−^(δ_1_) (P-O symmetric bending)
583–585	(PO_4_)^3−^(δ_2_) (P-O asymmetric bending)
950–965	(PO_4_)^3−^(ν_1_) (P-O symmetric stretching)
1030–1045	(PO_4_)^3−^(ν_2_) (P-O asymmetric stretching)

**Table 3:** The Raman shift and full-width half maxima (FWHM) at different O_2_/C_2_H_2_ ratios.

Sample	Raman Shift Frequency (cm^−1^)	FWHM
HA powder	962 ± 0	9.24
HA 2.6	958 ± 4	33.54
HA 3.0	958 ± 4	27.72
HA 3.3	958 ± 4	35.58
HA 3.7	960 ± 2	19.04

**Table 4:** Results of the X-ray phase analysis.

Sample	Detected Phases	Phase Content, mas. %	Lattice Parameters, nm	CSR Size, nm	d/d × 10^−3^
HA 2.6	HA [Ca_5_(PO_4_)_3_OH]	71	a = 0.93949 c = 0.68811	48	1
	TCP [Ca_3_(PO_4_)_2_]	29	a = 1.2987 b = 2.72012 c = 1.29328	18	0.7
HA 3.0	HA [Ca_5_(PO_4_)_3_OH]	73	a = 0.93949 c = 0.68897	59	1
	TCP [Ca_3_(PO_4_)_2_]	27	a = 1.29515 b = 2.73361 c = 1.29063	25	1
HA 3.3	HA [Ca_5_(PO_4_)_3_OH]	71	a = 0.93896 c = 0.68866	60	0.8
	TCP [Ca_3_(PO_4_)_2_]	29	a = 1.29492 b = 2.7380 c = 1.28901	18	0.7
HA 3.7	HA [Ca_5_(PO_4_)_3_OH]	75	a = 0.94128 c = 0.69045	59	1
	TCP [Ca_3_(PO_4_)_2_]	25	a = 12.8684 b = 27.3485 c = 12.8559	25	1

**Table 5:** Surface roughness of the coating.

Sample	S_a_ (μm)	S_q_ (μm)	S_v_ (μm)	S_t_ (μm)
HA 2.6	7.9	10.4	14.6	70
HA 3.0	11.1	14.3	25.8	82.1
HA 3.3	10.3	13.2	28	76.3
HA 3.7	9.5	12.3	23.5	66

where S_a_ (arithmetic mean height) is the average of the absolute deviations of surface heights from the mean plane in 3D, S_q_ (root mean square height) is the root mean square of the surface height deviations from the mean plane, S_v_ (maximum pit depth) is the maximum vertical distance between the deepest pit and the mean plane of the surface, S_t_ (total height of the surface) is the vertical distance between the highest peak and the deepest valley of the surface, representing the total range of height variation across the surface.

**Table 6:** Results of the tribological tests for coatings in dry conditions and Ringer’s solution at different O_2_/C_2_H_2_ ratios and for titanium (Grade 2).

Sample	In Dry Conditions		In Ringer’s Solution	
	Coefficient of Friction [μ]	Volumetric Wear Index × 10^−6^ [mm^3^/Nm]	Coefficient of Friction [μ]	Volumetric Wear Index × 10^−6^ [mm^3^/Nm]
HA 2.6	0.5 ± 0.29	741 ± 283	0.302 ± 0.014	150 ± 43
HA 3.0	0.87 ± 0.06	422 ± 73	0.287 ± 0.01	113 ± 34
HA 3.3	0.90 ± 0.06	331 ± 90	0.296 ± 0.01	108 ± 30
HA 3.7	0.85 ± 0.09	1230 ± 148	0.319 ± 0.014	152 ± 35
Grade 2	0.65 ± 0.04	734 ± 266	0.471 ± 0.014	630 ± 120

## Data Availability

The original contributions presented in the study are included in the article, further inquiries can be directed to the corresponding author.
